# Targeting Proliferating Cell Nuclear Antigen and Its Protein Interactions Induces Apoptosis in Multiple Myeloma Cells

**DOI:** 10.1371/journal.pone.0070430

**Published:** 2013-07-31

**Authors:** Rebekka Müller, Kristine Misund, Toril Holien, Siri Bachke, Karin M. Gilljam, Thea K. Våtsveen, Torstein B. Rø, Emanuele Bellacchio, Anders Sundan, Marit Otterlei

**Affiliations:** 1 Department of Cancer Research and Molecular Medicine, Norwegian University of Science and Technology, Trondheim, Norway; 2 APIM Therapeutics AS, Trondheim, Norway; 3 Research Laboratories, Bambino Gesú Childreńs Hospital, IRCCS, Rome, Italy; 4 K.G. Jebsen Center for Myeloma Research, Trondheim, Norway; Universita’ di Milano, Italy

## Abstract

Multiple myeloma is a hematological cancer that is considered incurable despite advances in treatment strategy during the last decade. Therapies targeting single pathways are unlikely to succeed due to the heterogeneous nature of the malignancy. Proliferating cell nuclear antigen (PCNA) is a multifunctional protein essential for DNA replication and repair that is often overexpressed in cancer cells. Many proteins involved in the cellular stress response interact with PCNA through the five amino acid sequence AlkB homologue 2 PCNA-interacting motif (APIM). Thus inhibiting PCNA’s protein interactions may be a good strategy to target multiple pathways simultaneously. We initially found that overexpression of peptides containing the APIM sequence increases the sensitivity of cancer cells to contemporary therapeutics. Here we have designed a cell-penetrating APIM-containing peptide, ATX-101, that targets PCNA and show that it has anti-myeloma activity. We found that ATX-101 induced apoptosis in multiple myeloma cell lines and primary cancer cells, while bone marrow stromal cells and primary healthy lymphocytes were much less sensitive. ATX-101-induced apoptosis was caspase-dependent and cell cycle phase-independent. ATX-101 also increased multiple myeloma cells’ sensitivity against melphalan, a DNA damaging agent commonly used for treatment of multiple myeloma. In a xenograft mouse model, ATX-101 was well tolerated and increased the anti-tumor activity of melphalan. Therefore, targeting PCNA by ATX-101 may be a novel strategy in multiple myeloma treatment.

## Introduction

Multiple myeloma (MM) is a cancer with clonal proliferation of malignant plasma cells that accounts for about 13% of hematological cancers. The malignant cells in early- and middle-stage disease are found in the bone marrow, suggesting a dependency on the bone marrow microenvironment [1]. The median survival has increased for MM patients following the introduction of new treatments such as bortezomib and thalidomide/lenalidomide [2]. Nevertheless, MM is considered to be an incurable disease with high relapse frequencies and thus new treatments are urgently needed. It has been suggested that therapy targeting single pathways may have limited benefits because of the high heterogeneity of MM [3].

Proliferating cell nuclear antigen (PCNA) is an essential protein in DNA replication and associated processes such as chromatin remodeling/epigenetics and DNA repair [4], [5]. It is frequently used as a marker of proliferation and it is often overexpressed in cancer cells [6]. In line with this, increased PCNA expression has been correlated with increased micro vessel density and disease activity in MM bone marrow biopsies [7]. Until recently, PCNA was regarded as a strictly nuclear protein; however, PCNA in the cytosol of differentiated neutrophils has been reported to be involved in apoptosis regulation [8]. Additionally, PCNA was found to be an inhibitor of natural cytotoxicity receptor NKp44 and to promote immune evasion of cancer cells [9]. Furthermore, proteomic analysis has suggested that PCNA is involved in coordination of glycolysis via direct interactions with six glycolytic enzymes in the cytoplasm [10]. Thus, PCNA likely has several functions outside the nucleus and beyond DNA replication and repair.

The functionality of PCNA in the cell depends on its ability to bind and recruit other proteins. PCNA has more than 400 potential protein interaction partners where the interactions are mediated via the two known protein-interacting sequences, the PCNA-interacting peptide (PIP)-box [11] and AlkB homologue 2 PCNA-interacting motif (APIM) (http://tare.medisin.ntnu.no/pcna/index.php) [12]. We have previously found that overexpressing an APIM-containing peptide rendered cancer cells hypersensitive against various chemotherapeutics. The molecular mechanism for this effect has heretofore not been fully elucidated, but is likely explained by the ability of the APIM-peptide to inhibit the interaction between PCNA and several of the more than 200 proteins containing APIM including DNA repair proteins [12], [13].

In general, many targeted therapies fail due to development of resistant cancer cell clones or activation of redundant pathways [14]–[16]. The use of several different agents successively or simultaneously to overcome resistance is probably a good strategy [16]. Targeting PCNA would fit well with such strategies due to its vital role in regulation of cellular homeostasis. By targeting PCNA with ATX-101, an APIM-containing cell-penetrating peptide, we induced apoptosis in MM cell lines and primary cells, and increased the sensitivity against the chemotherapeutic melphalan. Moreover, ATX-101 improved the efficacy of melphalan in a xenograft MM mouse model. Our data suggest that the effects of ATX-101 are mediated via its interaction with PCNA, and are therefore likely caused by inhibition of PCNA’s normal interaction with partners involved in stress response regulation.

## Materials and Methods

### Expression Constructs

Cloning of the fluorescently tagged expression constructs CFP-PCNA and hABH2 1-7-F4W-YFP (APIM-YFP) has been described [12], [17]. The PIP-YFP (RFC 1-24-YFP) construct was a kind gift from Dr. Emma Warbrick, University of Dundee, UK. Site-directed mutagenesis of the PCNA construct was done according to the manufacturer’s manual (QuikChange, Agilent Technologies, Santa Clara, CA, USA).

### Cell Penetrating Peptides

We ordered a series of peptides containing: the APIM consensus (R/K- F/W/Y- L/I/V/A- L/I/V/A- K/R) [12] - a linker of 1-4 amino acids- a SV40 NLS (KKKRK)- a linker of 1-4 amino acids- and three different cell-penetrating peptides: Hiv-TAT (RKKRRQRRR), penetratin (RQKIWFQNRRMKWKK) and R-rich (11 Arg residues) (Innovagen, Lund, Sweden). We tested a selection of these peptides for import and localization in cells by adding a fluorescent tag to the C-terminus (K(-Ahx-5-FAM)G). The peptides were next tested for biological activity in cell proliferation assays (MTT and colony forming assays). We added an acetyl to the N-terminal M of the peptide in order to increase the stability. An APIM-cell penetrating peptide comprising the following sequence was selected as the lead candidate: Ac-MDRWLVKWKKKRKIRRRRRRRRRRR and named ATX-101. In the mutant version of ATX-101, ATX-A, W4 is changed to A.

#### Cells

HeLa cells (cervical cancer, ATCC CCL-2) transiently and stably expressing fluorescently tagged proteins were prepared and cultured as described [12]. JJN-3, RPMI-8226, URVIN (MM), and U937 (histiocytic lymphoma) cells were grown in RPMI 1640 (Sigma-Aldrich, Schnelldorf, Germany) supplemented with 10% FCS (heat-inactivated for RPMI-8226 and URVIN), 2 mM glutamine (Sigma-Aldrich), 2.5 µg/ml amphotericin B (Sigma-Aldrich) and 100 µg/ml gentamicin (Invitrogen, Carlsbad, CA, USA). KJON and VOLIN (MM) cells were maintained in 5% and 10% heat-inactivated human serum (HS), respectively, (Blood Bank, St. Olav’s University Hospital, Trondheim, Norway) in RPMI 1640 and IL-6 (2 ng/mL). JJN-3 (ACC 541, German Collection of Microorganisms and Cell Cultures, Braunschweig, Germany) are a gift from J. Ball (University of Birmingham, United Kingdom) and RPMI-8226 (ATCC CCL-155) and U937 (ATCC CRL-1593.2) are from American Type Culture Collection (Manassas, VA, USA). URVIN, KJON, and VOLIN were established in-house [18]. Peripheral blood lymphocytes were isolated from A+ buffy coats (Blood Bank, St. Olav’s University Hospital) by density gradient centrifugation (Lymphoprep; Axis-Shield PoC, Oslo, Norway) and were maintained in RPMI 1640 supplemented with 2 mM glutamine, 100 µg/ml gentamicin and 5% heat-inactivated HS. All cells were cultured at 37°C in a humidified atmosphere of 5% CO_2_.

### Immunofluorescence

Cells were grown on poly-lysine coated glass bottom dishes and were stained as described [13] using antibody against (α) PCNA (PC10, Santa Cruz biotechnology Inc., Dallas, TX, USA) and Alexa fluor 532 goat α-mouse (Invitrogen). The nuclei were stained with DRAQ5 according to the manufacturer’s manual (eBioscience, San Diego, CA, USA).

### Confocal Imaging

Live HeLa cells were examined 16–24 h after transient transfection (by Fugene HD or X-tremeGENE HP [Roche, Oslo, Norway] according to the manufacturer’s recommendations) with the CFP/YFP fusion constructs. The fluorescent images were acquired using a Zeiss LSM 510 Meta laser scanning microscope equipped with a Plan-Apochromate 63×/1.4 oil immersion objective in the growth medium of the cell, with the stage heated to 37°C, using the Zeiss LSM 510 software. CFP was excited at λ = 458 nm and detected at λ = 470–500 nm and YFP was excited at λ = 514 nm and detected at λ = 530–600 nm or λ>560 nm. The fluorescently labeled ATX-101 and ATX-A were excited at λ = 488 nm and detected at λ = 505–530 nm in living HeLa cells directly after addition in serum-free growth medium. The immunofluorescently stained cells were excited at λ = 543 nm and detected at λ> = 560–615 nm in 2% FCS in PBS at RT. DRAQ5 was excited at λ = 633 nm and detected at λ>650 nm. The thickness of the slice was 1 µm. All images were acquired with consecutive scans to avoid bleed through. No image processing, except contrast and intensity adjustments, were performed.

### Fluorescence Resonance Energy Transfer (FRET) Analysis

FRET occurs if tags with spectral overlap (here: CFP and YFP) are less than 100 Å (10 nm) apart [19]. We detected FRET using the sensitized emission method, measuring acceptor (YFP) emission upon donor (CFP) excitation. FRET was scored when the intensity of emitted light from YFP after excitation of the CFP fluorochrome was stronger than the light emitted by CFP or YFP-tagged proteins alone, after excitation with the CFP laser (false FRET), given by the equation: FRET = I2–I1 (ID2/ID1) - I3 (IA2/IA3). I is mean intensity detected in the 3 different channels. ID1, D2, D3 and IA1, A2, A3 were determined for cells transfected with CFP and YFP constructs only, with same settings and same fluorescence intensities as co-transfected cells (I1, I2, I3). FRET >0 was normalized for expression levels using the equation: N_FRET_ = FRET/(I1×I3)1/2 [20], [21]. N_FRET_ was calculated from mean intensities within a region of interest representing replication foci containing more than 25 pixels where all pixels had intensities below 250. Channel 1 (CFP) and 3 (YFP) were measured as described for confocal imaging, and channel 2 (FRET) was excited with λ = 458 nm and detected at λ = 530–600 nm or λ>560 nm.

### Flow Cytometry

Cells were treated with different doses of ATX-101 alone and in combination with melphalan and were collected after the indicated time of continuous exposure. For measurement of the apoptotic cell population, the cells were stained with annexin V-Pacific Blue (Invitrogen) and propidium iodide to stain for dead cells, when noted, according to the manufacturer’s instructions. For cell cycle phase analysis cells were additionally stained with DRAQ5. Specific caspase activity was detected by FLICA Caspase Assay Kits (Immunochemistry Technologies LLC, Bloomington, MN, USA). All cells were analyzed by a FACSAria and the FACSDiva software (BD Biosciences, San Jose, CA, USA).

### Preparation of Cell Extract and Western Analysis

JJN-3 cells were seeded at 350 000 cells/ml and treated with ATX-101/melphalan overnight as indicated. RPMI-8226 cells were seeded and treated as JJN-3 for only 4 h. The cells were harvested and the pellet was resuspended in buffer 1 (10 mM Tris-HCl pH 8.0, 200 mM KCl, 1 mM DTT, 10 µl/ml Phosphatase Inhibitor Cocktail [PIC 1 and 2, Sigma-Aldrich] and 1×Complete Protease Inhibitor [Roche]). The same volume of buffer 2 (10 mM Tris-HCl pH 8.0, 200 mM KCL, 2 mM EDTA, 40% glycerol, 0.5% NP40, 1 mM DTT, 10 µl/ml PIC 1 and 2 and 1×Complete Protease Inhibitor) was added and incubated for 1.5 h at 4°C on a roller shaker. The cell extracts were centrifuged and separated on 4–12% Bis-Tris-HCl (NuPAGE, Invitrogen) gels. Proteins were detected by western blot as described [12] using α-caspase 3 (9662), α-cleaved caspase 8 (9748, Cell Signaling, Beverly, MA, USA), and α-beta-actin (Abcam, Cambridge, UK) as loading control.

### Immunoprecipitation

1 000 µg cell extract from JJN-3 cells were incubated with 5 µl α-PCNA-coupled beads (Abcam 18197) under constant rotation at 4°C overnight. The beads were washed 3 times with buffer 1, resuspended in LDS loading buffer (NuPAGE, Invitrogen) and 1 mM DTT and heated for 15 min at 70°C. Elutions were analyzed by western blot.

### Cell Survival Assay

Cells were seeded into 96-well plates and different doses of ATX-101 and chemotherapeutic drugs were added. Cells were exposed continuously and harvested every day for the next four days using the MTT (3-(4.5-Dimethylthiazol-2-yl)-2.5 diphenyl-tetrazolium bromide) assay as described [12]. The average from at least 4 wells was used to calculate cell survival.

### Primary Myeloma Cells

Fresh CD138 positive myeloma cells were isolated from bone marrow samples obtained from the Norwegian Myeloma Biobank using RoboSep automated cell separator and Human CD138 Positive Selection Kit (StemCell Technologies, Grenoble, France). The patient myeloma cells were grown in RPMI medium supplemented with 2% heat-inactivated HS and melphalan/ATX-101 as indicated. Cell viability was measured after 3 days using annexin V-FITC and propidium iodide staining (as described for Flow cytometry). All samples were run in duplicate. Bone marrow stromal cells (BMSC) were made by seeding the remaining mononuclear cells from the CD138 positive selection in culture flasks. The cells were grown in RPMI with 10% HS. After 3 days of culture, cells in suspension were removed, and the remaining adherent cells were expanded and split after about 10 days. After 3 weeks, cells from ten different patients were mixed to obtain standardized BMSC for use in co-culture experiments with myeloma cells.

### Co-culture Experiments

BMSC (2 500 cells/well) were plated in 96-well plates and allowed to adhere for ∼3 h, before addition of primary myeloma cells (5 000 cells/well). Drugs were added at the concentrations indicated. The cells were cultivated in RPMI medium supplemented with 2% HS in a total volume of 200 µl/well. Experiments were also performed with primary myeloma cells alone. All samples were run in duplicate. After 3 days incubation 1 µM YO-PRO-1 (Invitrogen) were added to the wells and the plate was incubated for 30 minutes at 37°C. 2.5 µM DRAQ5 were added to the wells 15 minutes before fluorescence was measured at a ScanR automated fluorescence microscope (Olympus, Hamburg, Germany). Acquired images were analyzed using ScanR Image Analysis software. Details on how the image acquisition and image analysis were performed will be presented in a separate method paper [22]. In brief, BMSC and myeloma cells were distinguished and gated based on the staining intensity and area of their nucleus. Viable cells were gated by a high intensity of DRAQ5 nuclear staining and low YO-PRO-1 staining.

### Mice Xenograft Study

Efficacy experiments using intra-peritoneal (i.p.) injections of vehicle, ATX-101, melphalan or a combination of melphalan and ATX-101 on subcutaneous RPMI-8226 human myeloma xenografts in NOD/SCID mice were performed by Crownbio (Changping Sector of Zhongguancum Scientific Park, No.21 Huonju Road, Changping District, Beijing, China). Ten tumor-bearing 8–9 weeks old NOD/SCID mice (22–28 g) in each group were observed in a 3-week treatment efficacy study during a 29-day in-life period after their mean tumor volume reached 129 mm^3^. Tumor volumes were measured twice per week in two dimensions using a caliper, and the volume is given in mm^3^ using the formula: V = 0.5 *a*×*b* ^2^ where *a* and *b* are the long and short diameters of the tumor, respectively. ATX-101 was given twice daily for 3 weeks (Bid×7×3, i.p.) at a dose of 13.5 mg/kg per dose, whereas melphalan was given once weekly for three weeks (i.p.) at a dose of 3 mg/kg. Of note, the chronic maximum tolerated dose i.p. of ATX-101 exceeds 25 mg/kg in SCID mice (unpublished data).

### Ethics Statement

The study on patient myeloma cells and the establishment of the cell lines KJON, URVIN, and VOLIN were approved by the Regional Committee for Medical and Health Research Ethics Central Norway (REC Central, permit numbers: REK 2011/2029 and REK 4.2007.933) and the patients had given written informed consent. The animal study was performed by Crownbio according to international regulations. The protocol and any amendment(s) or procedures involving the care and use of animals in this study were reviewed and approved by the Institutional Animal Care and Use Committee (IACUC) of Crownbio prior to conduct. During the study, the care and use of animals was conducted in accordance with the regulations of the Association for Assessment and Accreditation of Laboratory Animal Care (AAALAC). Animals that were observed to be in a continuing deteriorating condition or for which the tumor size exceeded 3000 mm^3^ were euthanized prior to death, or before reaching a comatose state. One animal (vehicle control group) was sacrificed on day 22.

## Results

### Design of ATX-101, a Cell-penetrating APIM-containing Peptide Targeting PCNA

**Figure 1:**
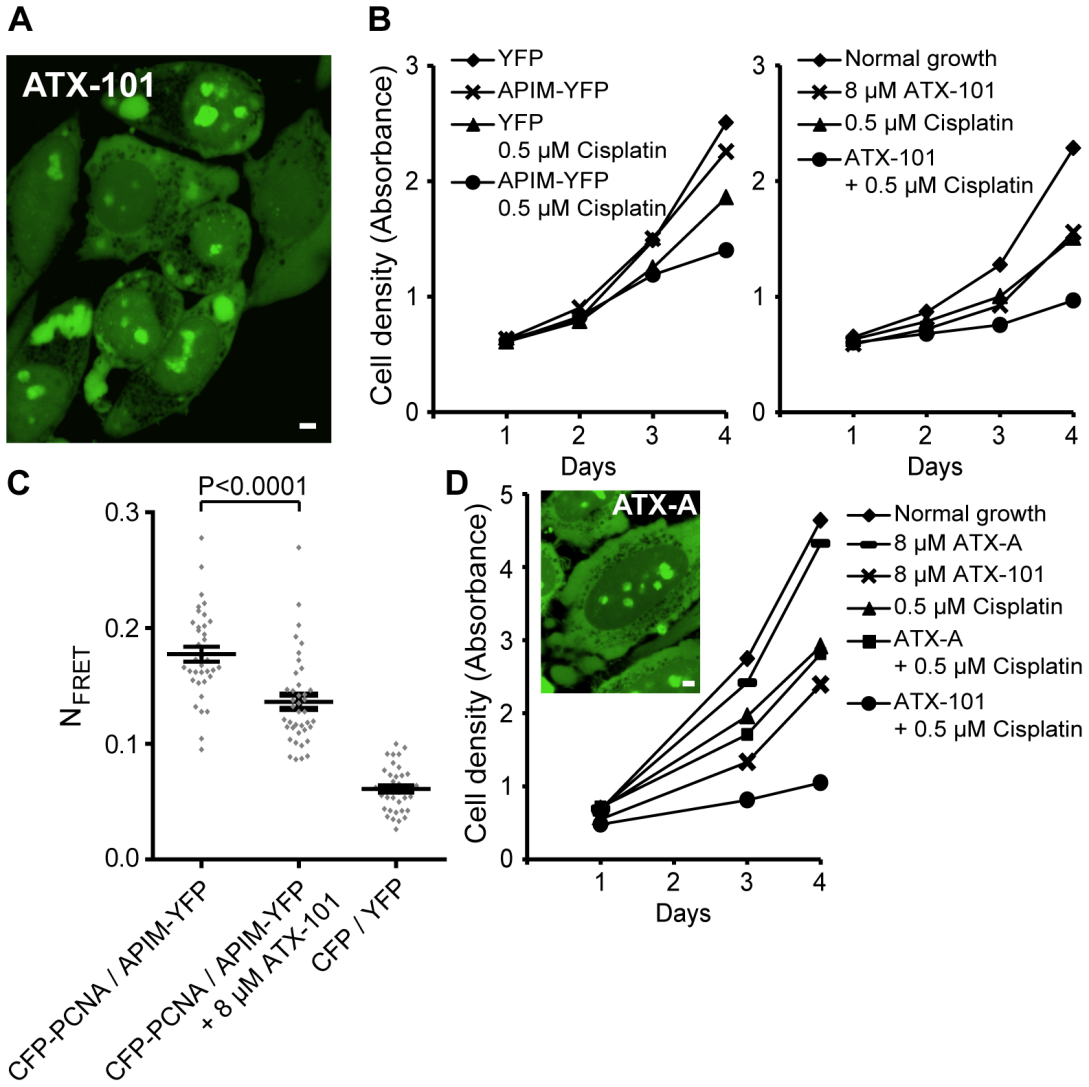
ATX-101, a cell-penetrating APIM-peptide, targets PCNA. (A) Confocal fluorescence image of live HeLa cells 2 minutes after addition of fluorescently tagged ATX-101. Bar, 5 µm. (B) Cell growth measured by MTT assay of HeLa cells stably expressing YFP and APIM-(hABH2 _1–7_ F4W)-YFP unexposed (♦ and×, respectively) and after continuous exposure to 0.5 µM cisplatin (▴ and •, respectively) (left panel) and parental HeLa cells unexposed (♦) and after continuous exposure to 8 µM ATX-101 (×), 0.5 µM cisplatin (▴), and combination of ATX-101 and cisplatin (•) (right panel). Data is from one representative experiment out of at least three. (C) Normalized FRET (N_FRET_) measurements in HeLa cells between CFP-PCNA and APIM-YFP without and in the presence of ATX-101. The cells were treated with 8 µM ATX-101 8 h after transient transfection and incubated for 16 h before the N_FRET_ measurements. CFP/YFP (vectors only) was used as background control. Data is from three independent experiments (mean ± SEM, n = 36–40). P-value was calculated by the unpaired Student’s t-test. (D) Cell growth measured by MTT assay of HeLa cells unexposed (♦) and after continuous exposure to 8 µM ATX-A (—), 8 µM ATX-101 (×), 0.5 µM cisplatin (▴), and combination of ATX-A or ATX-101 and cisplatin (▪ and •, respectively). The confocal image shows fluorescently tagged ATX-A in HeLa cells as in (A). Bar, 5 µm. Data is from one representative experiment out of three.

To identify peptides that could penetrate the cell membrane and target PCNA throughout the cell, we constructed a series of peptides containing the APIM consensus with a SV40 nuclear localization signal and different cell-penetrating peptide domains. We subsequently tested these peptides for cellular import and biological activity and selected one lead candidate, ATX-101. A fluorescently tagged version of ATX-101 showed that it was rapidly imported into the cells, where it localized to the cytosol, throughout the nucleoplasma, in nucleoli, and in small spots in the nucleoplasma (Figure 1A). We found that addition of ATX-101 to HeLa cells increased their sensitivity against the intra- and interstrand crosslinker cisplatin similarly to intracellularly expressed APIM-YFP (Figure 1B). This is in accordance with previous results for methyl methanesulfonate, carmustine, temozolomide, and mitomycin C treated HeLa cells [12]. APIM-YFP interacts directly with PCNA via the APIM sequence, and mutation in the conserved aromatic amino acid 4 in APIM (F/W/Y to A) abolished this interaction and the ability to sensitize cells to chemotherapeutics [12]. Thus, we examined whether the interaction between APIM-YFP and CFP-PCNA was affected by ATX-101 addition by measuring fluorescence resonance energy transfer (FRET) (Figure 1C). The addition of ATX-101 reduced the FRET level measured between APIM-YFP and CFP-PCNA, strongly indicating that ATX-101 impairs the APIM-PCNA interaction. These results suggest that the APIM sequence in ATX-101 is targeting PCNA because the only similarity between ATX-101 and APIM-YFP is the APIM sequence. To further support that PCNA is the cellular target of ATX-101, we designed a mutant cell-penetrating peptide version of ATX-101, ATX-A, in which only W4 in the APIM sequence of ATX-101 is substituted with A. ATX-A was imported into HeLa cells similarly to ATX-101, but it did not sensitize the cells to cisplatin (Figure 1D). This suggests that the sensitizing effect of ATX-101 is caused by PCNA targeting via the APIM sequence.

### ATX-101 Inhibits Cell Growth and Potentiates the Cytotoxicity of Melphalan in MM Cell Lines

**Figure 2:**
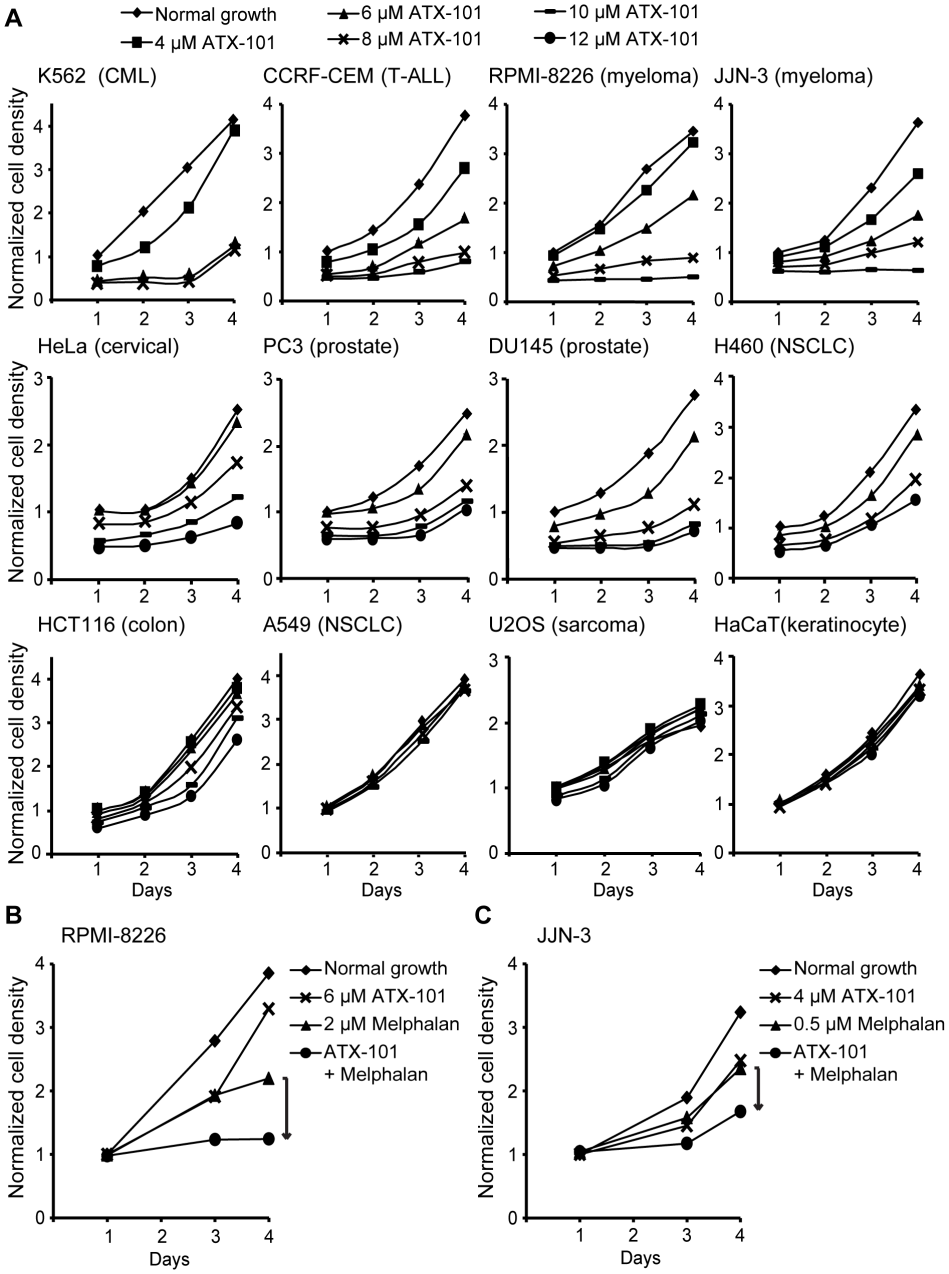
ATX-101 inhibits cell growth of cancer cell lines. (A) Cell growth after ATX-101 addition in different cell lines measured by MTT assay. K562 (chronic myelogenous leukemia), CCRF-CEM (T-lymphoblast, acute lymphocytic leukemia), RPMI-8226 and JJN-3 (MM), HeLa (cervical cancer), PC3 and DU145 (prostate cancer), H460 (non-small cell lung carcinoma), HCT116 (colorectal carcinoma), A549 (non-small cell lung carcinoma), U2OS (osteosarcoma) and HaCaT (spontaneously immortalized keratinocyte) cells were left unexposed (♦) and exposed to 4, 6, 8, 10, and/or 12 µM of ATX-101 (▪, ▴, ×, —, and •, respectively). (B and C) Cell growth measured by MTT assay of the MM cell lines RPMI-8226 and JJN-3, respectively, unexposed (♦) and after continuous exposure to 6 or 4 µM of ATX-101 (×), 2 or 0.5 µM melphalan (▴), and combination of ATX-101 and melphalan (•). (A–C) Data is normalized to cell growth from untreated cells on day 1 and from one representative experiment out of at least three.

Having established that ATX-101 is properly imported in HeLa cells and targets PCNA, we sought to identify ATX-101 sensitive cancer cell lines using proliferation assays. Different sensitivity against ATX-101 was detected in the different cancer cell lines, and hematological cancer cell lines were highly sensitive (Figure 2A).

The MM cell lines RPMI-8226 and JJN-3 were sensitive to ATX-101 either alone or in combination with melphalan, an interstrand crosslinking drug frequently used in MM treatment (Figures 2A–C). Furthermore, ATX-101 increased the sensitivity of these MM cell lines against other chemotherapeutics such as thalidomide, doxorubicin, vorinostat, azacitidine, and various kinase inhibitors (unpublished data). Similar results were found in the MM cell lines U266 and H929 (unpublished data).

### ATX-101 Induces Rapid Apoptosis in MM Cell Lines

**Figure 3:**
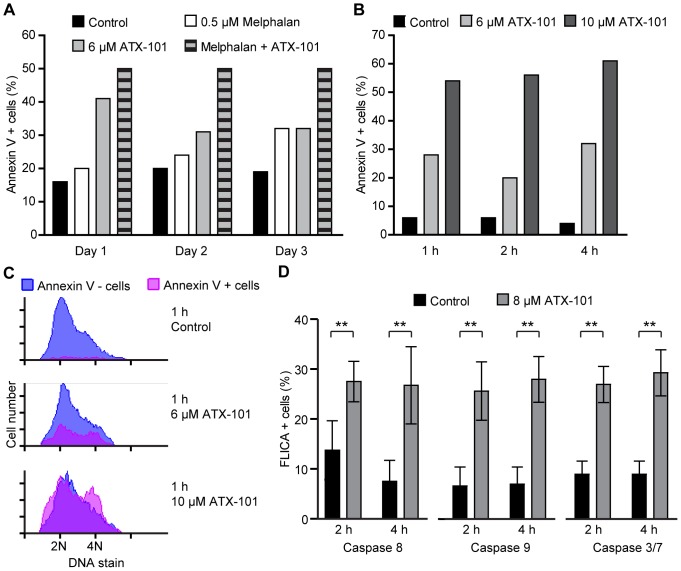
ATX-101 induces apoptosis in the MM cell line JJN-3. (A–C) Flow cytometric measurement of the apoptotic cell population by annexin V-Pacific Blue labeling. (A) JJN-3 cells treated with 6 µM ATX-101 and 0.5 µM melphalan alone or combined were incubated for 1, 2, and 3 days. Control cells were left unexposed. (B and C) JJN-3 cells treated with 6 and 10 µM ATX-101 were incubated for 1, 2, and 4 h. In addition to annexin V labeling, cells were stained with DRAQ5 for DNA profile. (C) The histograms show the cell cycle distribution of live (blue) and apoptotic (pink) cells after 1 h of ATX-101 treatments. (A–C) show data from representative experiments out of three. (D) Flow cytometric measurement of caspase 8, 9, and 3/7 activity by Fluorescent Labeled Inhibitor of Caspases (FLICA) assay. JJN-3 cells were left unexposed and exposed to 8 µM ATX-101 for 2 and 4 h before the FLICA probe was added for staining. The FLICA probe binds irreversible only to the activated caspase and labels apoptotic cells. Data is from four independent experiments for caspase 8 activity and three independent experiments for caspase 9 and 3/7 activity (mean ± SD, ** P < 0.01, Student’s t-test).

Next, we examined whether ATX-101 actively induced apoptosis. We observed the apoptotic cell population of JJN-3 cells continuously exposed to ATX-101 and/or melphalan over 3 days by annexin-V staining. ATX-101 induced apoptosis as a single agent in addition to increasing melphalan-induced apoptosis (Figure 3A). Notably, ATX-101 and melphalan alone showed a temporally different induction of apoptosis: the pro-apoptotic effect of ATX-101 was most pronounced on day 1, whereas melphalan increased the apoptotic cell population over time. ATX-101 had an increasing effect on melphalan-induced apoptosis at all days. Furthermore, we found that apoptosis was induced even 1 h after ATX-101 addition, and that apoptotic cells were found in all phases of the cell cycle (Figures 3B and C). We included propidium iodide controls for membrane permeability to check whether the cell-penetrating properties of ATX-101 damaged the cell membrane leading to false positives. We found no increase in propidium iodide uptake at these time points (unpublished data).

We next tested whether the rapid ATX-induced apoptosis was mediated by caspases. ATX-101 induced caspase 8, 9 and 3/7 activation within 2 h as assessed by specific FLICA caspase assays (Figure 3D). Thus, our results show that ATX-101 induces rapid apoptosis via caspase-dependent mechanisms in MM cells.

### APIM and PIP-box Peptides have Overlapping Binding Sites on PCNA, Suggesting that Targeting PCNA with ATX-101 Induces Caspase Cleavage by Inhibiting PCNA-procaspase Interactions

**Figure 4:**
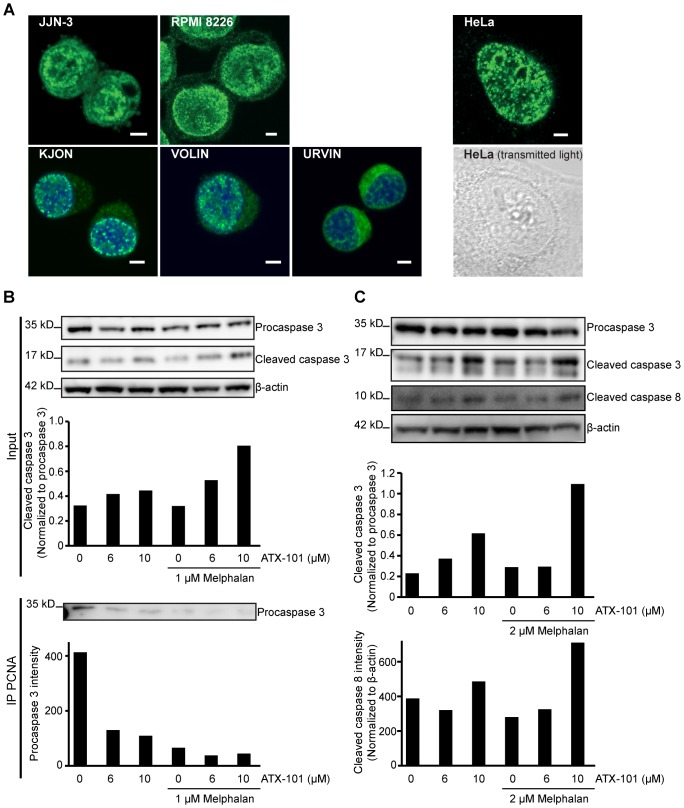
MM cell lines have PCNA in their cytosol. (A) Confocal fluorescence images of immunofluorescently stained PCNA (green) in JJN-3, RPMI-8226 cells and three new in-house made MM cell lines (KJON, VOLIN, and URVIN). DRAQ5 was used for nuclear staining (blue) of KJON, VOLIN, and URVIN. HeLa cells were used as control for low levels of PCNA staining in the cytosol (fluorescence image of PCNA and transmitted light image). Bar, 5 µm. (B) Western blot analysis of caspase 3 cleavage (Input, upper panel) and co-immunoprecipitation of procaspase 3 using α-PCNA beads (IP PCNA, lower panel) from JJN-3 cells after overnight treatment with 6 and 10 µM ATX-101 alone or in combination with 1 µM melphalan. The bar graphs show the quantification from the blot above. Cleaved caspase 3 in the input samples (upper panel) was corrected for loading differences (β-actin) and normalized to procaspase 3. (C) Western blot analysis of caspase 3 and caspase 8 cleavage in RPMI-8226 cells after 4 h treatment with 6 and 10 µM ATX-101 alone or in combination with 2 µM melphalan. The bar graphs show the quantification of cleaved caspase 3 normalized to procaspase 3 after correction for loading differences (β-actin) (upper graph) and cleaved caspase 8 normalized to β-actin (lower graph). (B and C) show results from representative experiments out of at least three.

Our mechanistic studies on ATX-101 (Figure 1) suggest that the APIM sequence in ATX-101 targets PCNA. Interestingly, it has been reported that PCNA is found in the cytosol of differentiated neutrophils where it exerted an anti-apoptotic effect by directly interacting with procaspase 3, 8, 9, and 10 [8]. Thus first, we examined the PCNA content in different compartments in MM cells. We tested RPMI-8226, JJN-3, and in-house prepared MM cell lines (KJON, URVIN, and VOLIN). All cell lines contained higher PCNA levels in the cytosol than HeLa cells (Figure 4A). Next, we immunoprecipitated PCNA from JJN-3 cell extracts and analyzed the precipitate for procaspase 3. We verified that procaspase 3 co-immunoprecipitates with PCNA. Additionally, we found that less procaspase 3 was co-immunoprecipitated in cells treated with ATX-101 (Figure 4B, lower panel). We also verified that ATX-101 treatment induced procaspase 3 and 8 cleavage by western analysis (Figures 4B and C), further supporting a caspase-dependent apoptosis.

**Figure 5:**
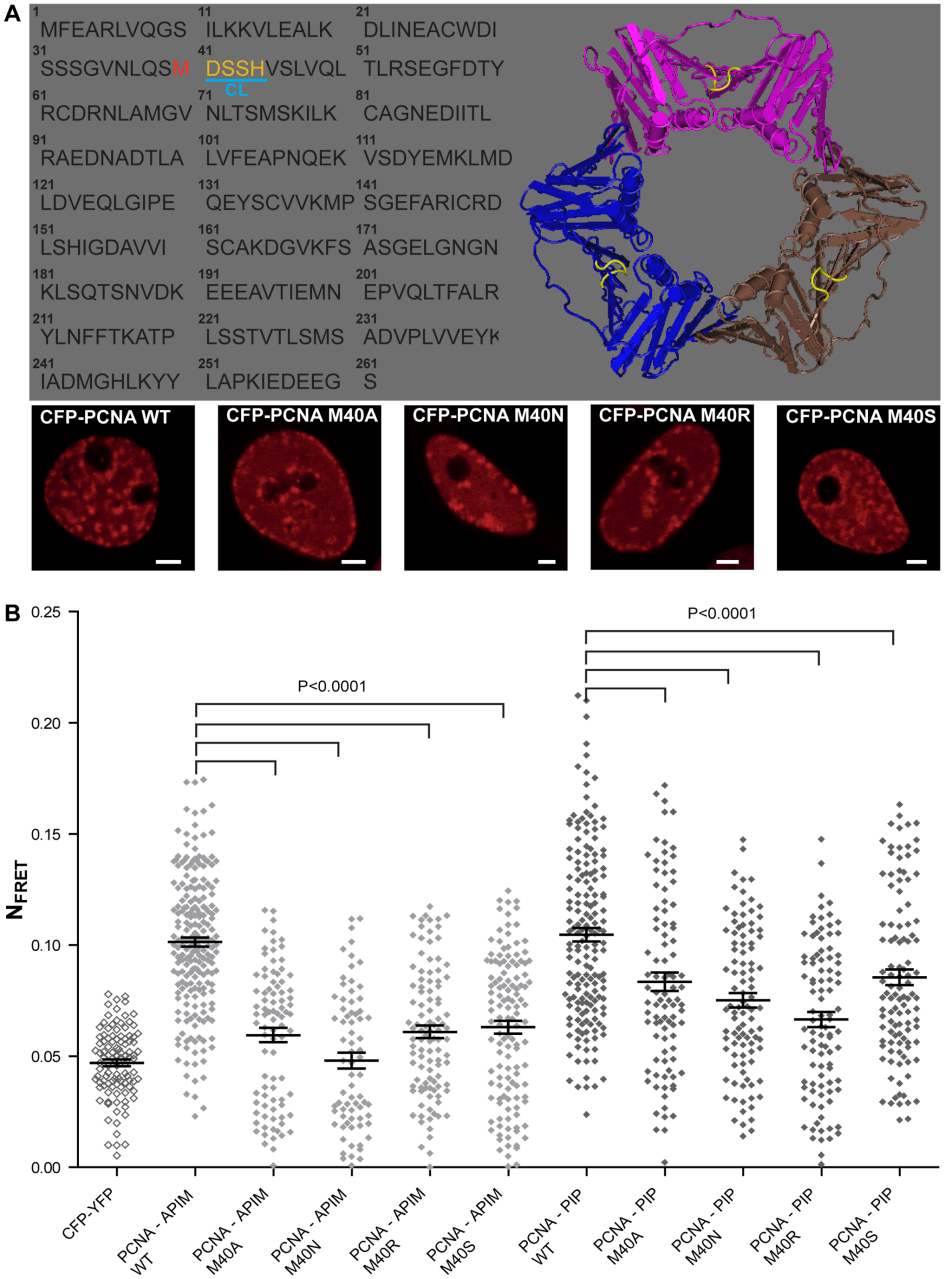
APIM and PIP-box peptides have overlapping binding site on PCNA. (A) Protein sequence and structural model of PCNA (PDB entry 1vym) with M40 highlighted in red and the center loop (CL) in yellow (upper panel). Live cell (HeLa) confocal fluorescence images of CFP-PCNA wild type (WT) and CFP-PCNA M40 mutants. Bar, 5 µm (lower panel). (B) Normalized FRET (N_FRET_) measurements between WT and mutated CFP-PCNA M40/APIM-YFP (light grey diamonds, PCNA WT−/PCNA M40A−/PCNA M40N−/PCNA M40R−/PCNA M40S- APIM) and WT and mutated CFP-PCNA M40/PIP-YFP (dark grey diamonds, PCNA WT−/PCNA M40A−/PCNA M40N−/PCNA M40R/PCNA M40S- PIP). CFP/YFP (vectors only) was used as background control (open diamonds). Data is from three independent experiments (mean ± SEM, n = 72–214). P-values were calculated by the unpaired Student’s t-test.

Witko-Sarsat and colleagues reported that a peptide containing the PIP-box inhibited the interaction between PCNA and the procaspases, and thereby induced apoptosis [8]. The rapid apoptosis observed after ATX-101 addition may suggest that the APIM-peptide has a similar mechanism of action. We therefore examined whether the APIM sequence bound to the same site on PCNA as the PIP-box. The interaction site of the PIP-box on PCNA is known, and the PIP-box containing protein FEN-1 has been co-crystallized with PCNA [23]. The PIP-box in FEN-1 is embedded in a hydrophobic pocket in PCNA close to the center loop (CL) (yellow in Figure 5A). In order to examine whether the APIM-peptide used shared interaction site on PCNA with the PIP-box, we selected one amino acid in this hydrophobic pocket of PCNA for mutation (Met (M) 40 adjacent to CL). We mutated M40 in PCNA to Ala (A), Asn (N), Ser (S), and Arg (R). The intracellular localization of these mutated PCNAs was similar to wild type PCNA, supporting functionality of the mutant proteins (Figure 5A, lower panel). Thus, we were able to measure FRET between the different mutated CFP-PCNAs and APIM-YFP, as well as between CFP-PCNAs and PIP-YFP. A reduction in FRET is observed when the interaction between APIM-PCNA or PIP-PCNA is impaired by the mutation. We found that FRET between both APIM-PCNA and PIP-PCNA were reduced when M40 was mutated (Figure 5B). This suggests that both the APIM sequence and the PIP-box interact with the hydrophobic pocket of PCNA. Thus, a likely mechanism of action for ATX-101 in MM cells is that it induces caspase-dependent apoptosis by inhibiting the interaction of the procaspases with PCNA similarly to what is reported for the PIP-peptide [8].

### ATX-101 Induces Apoptosis in a Cancer Cell Specific Manner

**Figure 6:**
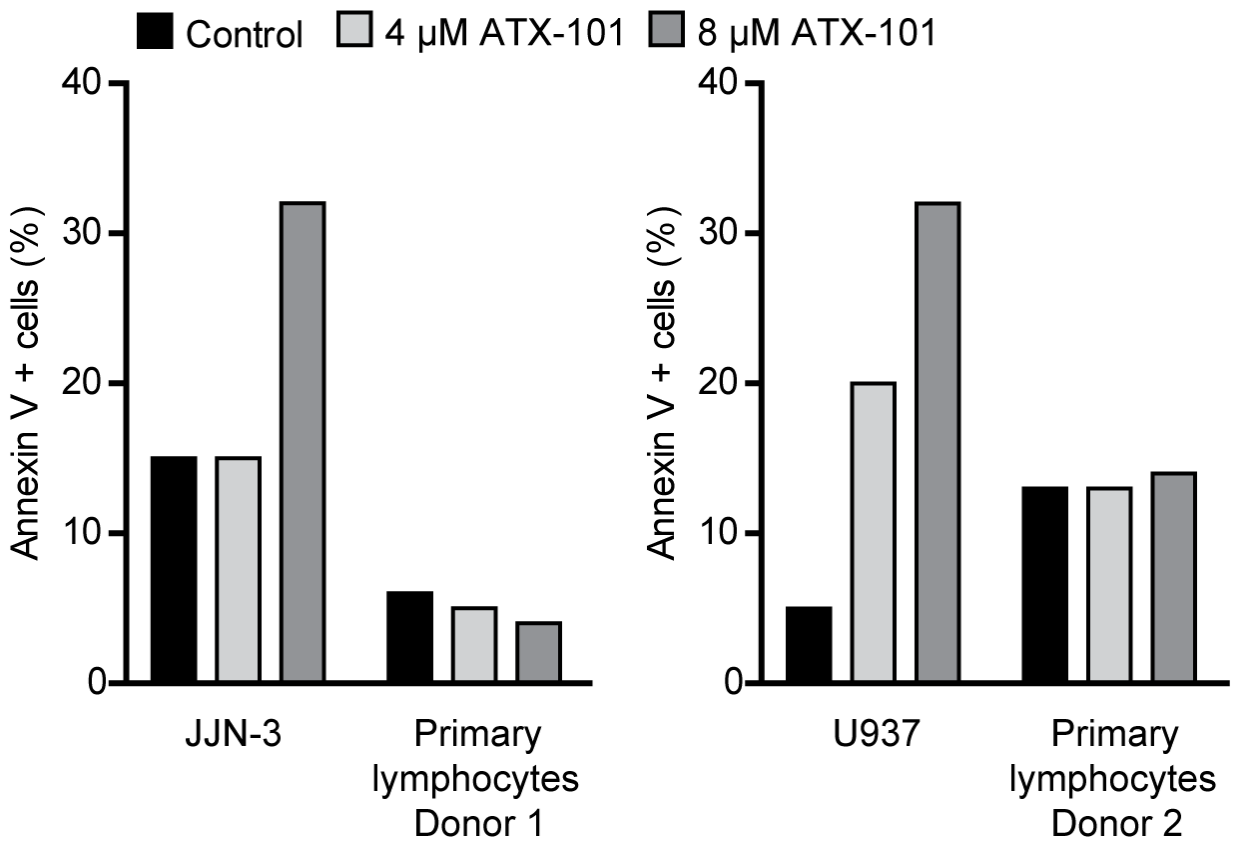
ATX-101 induces cancer cell specific apoptosis. Flow cytometric measurement of the apoptotic cell population by annexin V-Pacific Blue labeling. JJN-3 cells were treated with 4 and 8 µM ATX-101 for 2 h (left panel), and U937 cells were treated for 24 h (right panel). Lymphocytes freshly isolated from buffy coats (from blood donors) treated in parallel with JJN-3 and U937 are included as controls. Data is from representative experiments out of two.

We found that hematological cancer cell lines were generally more sensitive to ATX-101 as a single agent than other cancer cell lines (Figure 2A). To examine the pro-apoptotic effect of ATX-101 on healthy hematological cells, we compared ATX-101-induced apoptosis in JJN-3 and the leukemic monoblast cell line U937, with lymphocytes from healthy donors. We found that the cancer cell lines were more sensitive to ATX-101 than primary lymphocytes and monocytes (Figure 6 and unpublished data, respectively). These results indicate that ATX-101 induces apoptosis preferentially in cancer cell lines.

### ATX-101 Induces Apoptosis in MM Cells *ex vivo*

**Figure 7:**
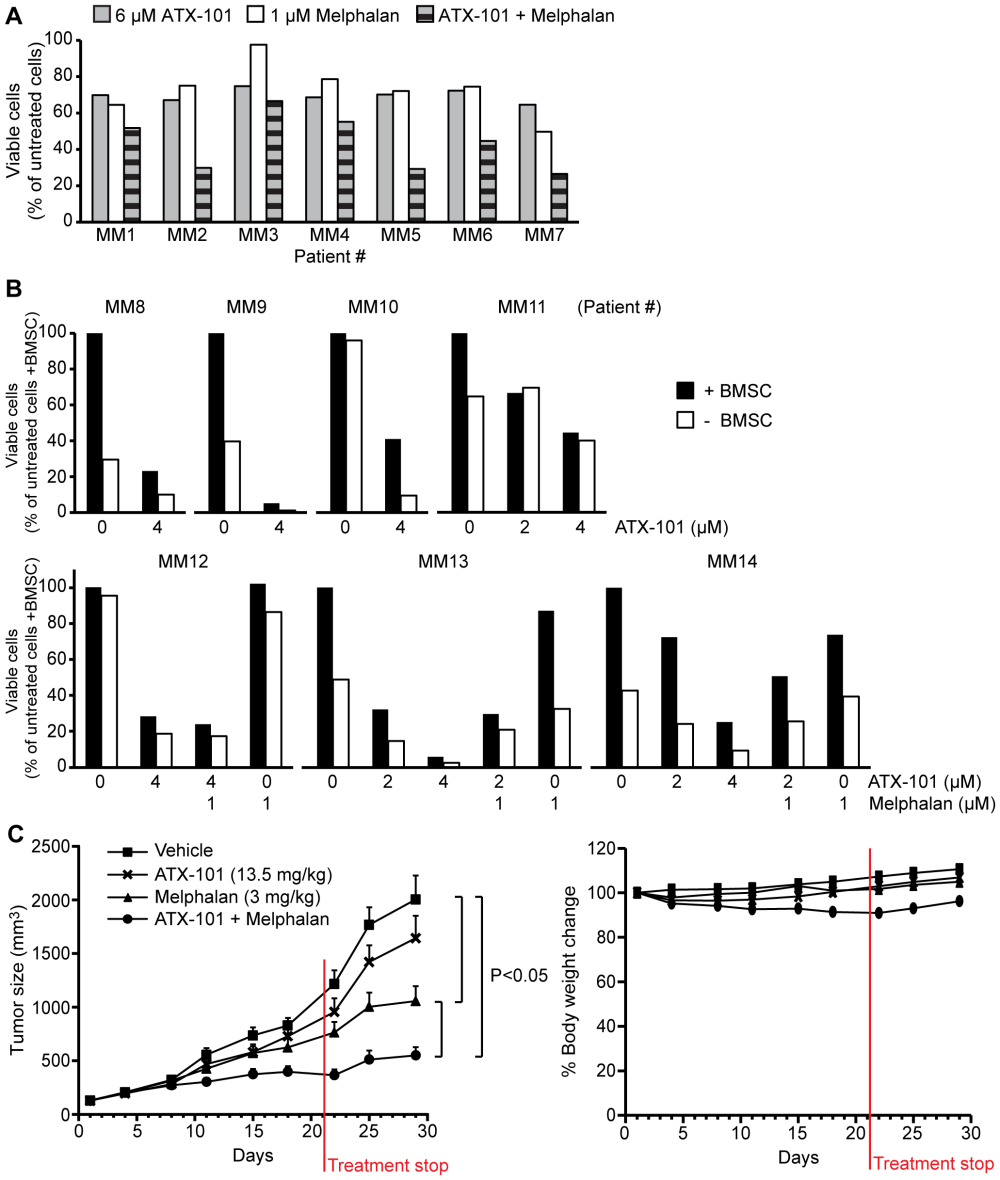
ATX-101 induces apoptosis in primary MM cells and increases the efficacy of melphalan *in vivo*. (A) Flow cytometric measurement of the viable cell population by annexin V-FITC and propidium iodide labeling of cancer cells isolated from seven MM patients (MM1–7) after treatment with 6 µM ATX-101 and 1 µM melphalan alone or in combination for 3 days. Data is normalized to the viable cell population of untreated patient cells after 3 days (100% viable cells). (B) Fluorescence microscopical measurement of the viable cell population by DRAQ5 and YO-PRO-1 staining of cancer cells from seven MM patients (MM8–14) cultured alone and in presence of BMSC. The cells were treated with 2 and/or 4 µM ATX-101 alone for 3 days (MM8-11). MM12 -14 were also treated with 1 µM melphalan alone and in combination with ATX-101. Data is normalized to the viable cell population of untreated patient cells in presence of BMSC after 3 days (100% viable cells). (C) NOD/SCID mice bearing subcutaneous RPMI-8226 tumors were treated with vehicle (▪), 13.5 mg/kg ATX-101 (×), 3 mg/kg melphalan (▴), and combination of ATX-101 and melphalan (•). Tumor volume (left panel) and body weight changes (right panel) are shown as mean ± SD (n = 10 mice/group). P-values were calculated by the ANOVA test.

Unlike MM cell lines, primary MM cells rarely proliferate *in vitro*. Thus, we examined the effect of *ex vivo* ATX-101 treatment on primary MM cells both alone and in combination with melphalan. We found that ATX-101 induced apoptosis as a single agent, similar to what we observed in MM cell lines. Additionally, we detected an increase in the efficacy of melphalan in combination with ATX-101 in some cases (Figure 7A). In all of the 19 patient samples tested (14 of which are displayed in Figures 7A and B) an increase in apoptosis was observed after addition of ATX-101 doses between 2–6 µM.

The bone marrow provides a protective microenvironment for the MM cells, and resistance toward anti-cancer drugs can be mediated through supportive stromal cells and/or extracellular matrix in the bone marrow [24], [25]. However, in our BMSC co-culture apoptosis assay ATX-101 also efficiently induced apoptosis in primary MM cells (Figure 7B). Under the conditions used in these experiments less than 20% of apoptotic cells were detected in the BMSC population (unpublished data), further supporting a cancer cell specificity of ATX-101. The efficacy of ATX-101 is comparable to melphalan and bortezomib at similar molar concentration using this assay (Figure 7B and unpublished data, respectively). These results suggest that the pro-apoptotic effect of ATX-101 is not restricted to highly proliferating cancer cell lines but can also be observed in *ex vivo* assays of patient-derived non- or slowly proliferating MM cells.

### ATX-101 Potentiates the Efficacy of Melphalan in a Xenograft MM Mouse Model

To further corroborate our *in vitro* and *ex vivo* results, we sought to establish the therapeutic effect of ATX-101 in an *in vivo* xenograft MM mouse model. ATX-101 showed no significant efficacy as a single agent, but it improved the efficacy of melphalan significantly (Figure 7C). ATX-101 showed no toxic effects alone or in combination with melphalan as measured by body weight changes, suggesting that the treatment was well-tolerated (Figure 7C). These results confirm our *in vitro* and *ex vivo* data, and support the cancer cell specific action of ATX-101.

## Discussion

In this study we have shown that the multifunctional protein PCNA is a drug target of interest in MM treatment. The APIM-comprising peptide ATX-101 is capable of inducing rapid apoptosis as a single agent and increasing the cytotoxic effect of melphalan in MM cell lines, primary cells, and a xenograft mouse model. ATX-101 likely functions via several mechanisms because many proteins involved in stress responses contain the APIM sequence (http://tare.medisin.ntnu.no/pcna/index.php) and ATX-101 could potentially inhibit any of their interactions with PCNA.

Our data shows that ATX-101 induces rapid caspase-dependent apoptosis (within 1 h) and also increases the efficacy of melphalan up to four days after addition. This suggests that ATX-101 acts along at least two different pathways in MM cells: a direct and rapid apoptosis-inducing pathway and a more indirect pathway that is possibly dependent upon a reduced ability to repair melphalan-induced DNA damage. The basis for the latter is that DNA repair proteins involved in repair of melphalan-induced DNA damage have verified functional APIM sequences (RAD51B, topoisomerase II α, xeroderma pigmentosum group A) [12], [13]. In addition, ZRANB3, a central protein involved in restarting arrested replication forks, has a functional APIM sequence [26].

The rapid induction of apoptosis by ATX-101 could be explained by an inhibition of the binding between cytosolic PCNA and procaspases leading to direct caspase activation, similar to what was found in neutrophils by Witko-Sarsat *et al.* [8]; however, ATX-101 did not induce apoptosis in normal lymphocytes, monocytes, or BMSC, although monocytes showed similar cytosolic PCNA levels as MM cell lines (unpublished data). Thus, there seems to be some specificity toward malignant cells. Interestingly, we previously observed that the PCNA that was immunoprecipitated with an APIM-peptide had a different isoelectric distribution than the total PCNA in the cell, suggesting that the PCNA interacting with APIM-peptides has specific post-translational modifications [12]. The exact nature of these post-translational modifications remains elusive. Several proteins essential for replication contain the PIP-box (e.g. pol δ, RFC, FEN-1), while many proteins involved in stress related processes contain the APIM sequence [5], [12], [26]. It has been demonstrated that certain cancer cells are stressed as a consequence of their genomic mutations, and it is thus possible that the total PCNA in these cells contains more of a modified PCNA with high affinity for APIM-peptides that could sensitize these cancer cells to ATX-101 treatment. In accordance with the apoptosis-inducing activity of the PIP-box-peptide detected by Witko-Sarsat and colleagues [8], all attempts to make stable U2OS cells overexpressing a PIP-box-peptide fused to YFP were unsuccessful [27]; however, we found that U2OS cells expressing an APIM-YFP fusion protein are viable and have normal growth rates (unpublished data). U2OS cells are also tolerant to ATX-101 treatment (Figure 2A).

Thus even though our data suggests that APIM- and PIP-box-peptides have an overlapping binding site on PCNA, their binding to PCNA likely involves multilayered regulatory mechanisms, including different post-translational modifications and different affinities between and within the two PCNA-interaction motifs (regulation of PIP-PCNA interactions reviewed in [5]), that could partly explain the broader cytotoxicity of PIP-box peptides compared to our APIM-peptide ATX-101. Many different cellular functions are regulated by PCNA at different sites in the cell, e.g. in cytosol, replication foci, and at sites of DNA repair. There is also increasing evidence for different post-translational modifications on PCNA. During replication the PCNA molecules at the front of the replication fork carrying the replicative polymerase likely have either different or no post-translational modifications than the PCNA molecules interacting with proteins involved in arrested replication fork restart, post-replicative DNA repair, or chromatin remodeling [12], [13], [26], [28]–[31].

Importantly, ATX-101 treatment rapidly induced apoptosis in all cell cycle phases. This is mechanistically different from two small molecules that have recently been reported to bind PCNA and inhibit cancer cell growth by interfering with PCNA’s function during replication [32], [33]. That ATX-101 also rapidly induced cancer cell apoptosis in G1-phase, i.e. independent of replication, makes it a very good candidate for further development. Many drug candidates fail in Phase II after promising pre-clinical results partly because their activity is dependent on a high proliferation rate in the target cells [34]. Our data on primary MM cells, alone and in co-culture with BMSC, together with the data in the xenograft mouse model clearly support the pro-apoptotic cancer-specific activity of ATX-101. Analogous to the new role of PCNA as an inhibitor of the natural cytotoxicity receptor on natural killer cells [9], ATX-101 may interfere with the MM cell-BMSC interaction in addition to directly targeting the cancer cells. ATX-101 was well tolerated in mice suggesting that there is a significant therapeutic window.

Like most other cancers MM develops following a variety of different genetic aberrations. Recent studies involving whole genome sequencing in MM patients describe this heterogeneity and even point to the existence of multiple cancer cell clones with individual oncogenetic signatures within the same patient [14], [15]. Therefore, developing a curative treatment for MM patients would likely imply targeting several different signaling pathways and cellular functions simultaneously. The drugs currently used against MM have different modes of action: Thalidomide/lenalidomide has immunomodulatory and anti-angiogenic properties, bortezomib inhibits proteasomal degradation, and melphalan is a DNA damage-inducing alkylating agent. On the contrary, ATX-101 targets PCNA’s protein interaction via APIM and thus probably impairs several different functions such as regulation of apoptosis, DNA repair, chromatin remodeling/epigenetic changes, and regulation of several different signal transduction pathways simultaneously [12], [13]. This makes ATX-101 a promising new compound for the treatment of MM.

## References

[1] Palumbo A, Anderson K (2011) Multiple myeloma. N Engl J Med 364: 1046–1060. 21410373 10.1056/NEJMra1011442

[2] Kumar SK, Rajkumar SV, Dispenzieri A, Lacy MQ, Hayman SR, et al (2008) Improved survival in multiple myeloma and the impact of novel therapies. Blood 111: 2516–2520. 17975015 10.1182/blood-2007-10-116129 PMC2254544

[3] Avet-Loiseau H, Magrangeas F, Moreau P, Attal M, Facon T, et al (2011) Molecular Heterogeneity of Multiple Myeloma: Pathogenesis, Prognosis, and Therapeutic Implications. J Clin Oncol 29: 1893–1897. 21482986 10.1200/JCO.2010.32.8435

[4] Moldovan GL, Pfander B, Jentsch S (2007) PCNA, the maestro of the replication fork. Cell 129: 665–679. 17512402 10.1016/j.cell.2007.05.003

[5] Mailand N, Gibbs-Seymour I, Bekker-Jensen S (2013) Regulation of PCNA-protein interactions for genome stability. Nat Rev Mol Cell Biol 14: 269–282. 23594953 10.1038/nrm3562

[6] Stoimenov I, Helleday T (2009) PCNA on the crossroad of cancer. Biochem Soc Trans 37: 605–613. 19442257 10.1042/BST0370605

[7] Alexandrakis MG, Passam FH, Pappa CA, Dambaki C, Sfakiotaki G, et al (2004) Expression of proliferating cell nuclear antigen (PCNA) in multiple myeloma: Its relationship to bone marrow microvessel density and other factors of disease activity. Int J Immunopathol Pharmacol 17: 49–56. 10.1177/039463200401700107 15000866

[8] Witko-Sarsat V, Mocek J, Bouayad D, Tamassia N, Ribeil JA, et al (2010) Proliferating cell nuclear antigen acts as a cytoplasmic platform controlling human neutrophil survival. J Exp Med 207: 2631–2645. 20975039 10.1084/jem.20092241 PMC2989777

[9] Rosental B, Brusilovsky M, Hadad U, Oz D, Appel MY, et al (2011) Proliferating Cell Nuclear Antigen Is a Novel Inhibitory Ligand for the Natural Cytotoxicity Receptor NKp44. J Immunol 187: 5693–5702. 22021614 10.4049/jimmunol.1102267 PMC3269963

[10] Naryzhny SN, Lee H (2010) Proliferating cell nuclear antigen in the cytoplasm interacts with components of glycolysis and cancer. FEBS Lett 584: 4292–4298. 20849852 10.1016/j.febslet.2010.09.021

[11] Warbrick E (1998) PCNA binding through a conserved motif. Bioessays 20: 195–199. 9631646 10.1002/(SICI)1521-1878(199803)20:3<195::AID-BIES2>3.0.CO;2-R

[12] Gilljam KM, Feyzi E, Aas PA, Sousa MM, Muller R, et al (2009) Identification of a novel, widespread, and functionally important PCNA-binding motif. J Cell Biol 186: 645–654. 19736315 10.1083/jcb.200903138 PMC2742182

[13] Gilljam KM, Muller R, Liabakk NB, Otterlei M (2012) Nucleotide Excision Repair Is Associated with the Replisome and Its Efficiency Depends on a Direct Interaction between XPA and PCNA. PLoS One 7: e49199. 23152873 10.1371/journal.pone.0049199 PMC3496702

[14] Keats JJ, Chesi M, Egan JB, Garbitt VM, Palmer SE, et al (2012) Clonal competition with alternating dominance in multiple myeloma. Blood 120: 1067–1076. 22498740 10.1182/blood-2012-01-405985 PMC3412330

[15] Egan JB, Shi CX, Tembe W, Christoforides A, Kurdoglu A, et al (2012) Whole-genome sequencing of multiple myeloma from diagnosis to plasma cell leukemia reveals genomic initiating events, evolution, and clonal tides. Blood 120: 1060–1066. 22529291 10.1182/blood-2012-01-405977 PMC3412329

[16] Duncan JS, Whittle MC, Nakamura K, Abell AN, Midland AA, et al (2012) Dynamic Reprogramming of the Kinome in Response to Targeted MEK Inhibition in Triple-Negative Breast Cancer. Cell 149: 307–321. 22500798 10.1016/j.cell.2012.02.053 PMC3328787

[17] Aas PA, Otterlei M, Falnes PO, Vagbo CB, Skorpen F, et al (2003) Human and bacterial oxidative demethylases repair alkylation damage in both RNA and DNA. Nature 421: 859–863. 12594517 10.1038/nature01363

[18] Holien T, Vatsveen TK, Hella H, Waage A, Sundan A (2012) Addiction to c-MYC in multiple myeloma. Blood 120: 2450–2453. 22806891 10.1182/blood-2011-08-371567

[19] Matyus L (1992) Fluorescence resonance energy transfer measurements on cell surfaces. A spectroscopic tool for determining protein interactions. J Photochem Photobiol B 12: 323–337. 1578295 10.1016/1011-1344(92)85039-w

[20] Otterlei M, Bruheim P, Ahn B, Bussen W, Karmakar P, et al (2006) Werner syndrome protein participates in a complex with RAD51, RAD54, RAD54B and ATR in response to ICL-induced replication arrest. J Cell Sci 119: 5137–5146. 17118963 10.1242/jcs.03291

[21] Xia Z, Liu Y (2001) Reliable and global measurement of fluorescence resonance energy transfer using fluorescence microscopes. Biophys J 81: 2395–2402. 11566809 10.1016/S0006-3495(01)75886-9 PMC1301710

[22] Misund K, Baranowska KA, Holien T, Rampa C, Klein DC, et al.. (2013) A Method for Measurement of Drug Sensitivity of Myeloma Cells Co-cultured with Bone Marrow Stromal Cells. J Biomol Screen. 10.1177/1087057113478168 23446700

[23] Sakurai S, Kitano K, Yamaguchi H, Hamada K, Okada K, et al (2005) Structural basis for recruitment of human flap endonuclease 1 to PCNA. EMBO J 24: 683–693. 15616578 10.1038/sj.emboj.7600519 PMC549611

[24] Azab AK, Runnels JM, Pitsillides C, Moreau AS, Azab F, et al (2009) CXCR4 inhibitor AMD3100 disrupts the interaction of multiple myeloma cells with the bone marrow microenvironment and enhances their sensitivity to therapy. Blood 113: 4341–4351. 19139079 10.1182/blood-2008-10-186668 PMC2676090

[25] Meads MB, Gatenby RA, Dalton WS (2009) Environment-mediated drug resistance: a major contributor to minimal residual disease. Nat Rev Cancer 9: 665–A674. 19693095 10.1038/nrc2714

[26] Ciccia A, Nimonkar AV, Hu Y, Hajdu I, Achar YJ, et al (2012) Polyubiquitinated PCNA Recruits the ZRANB3 Translocase to Maintain Genomic Integrity after Replication Stress. Mol Cell 47: 396–409. 22704558 10.1016/j.molcel.2012.05.024 PMC3613862

[27] Warbrick E (2006) A functional analysis of PCNA-binding peptides derived from protein sequence, interaction screening and rational design. Oncogene 25: 2850–2859. 16407840 10.1038/sj.onc.1209320 PMC2699888

[28] Lehmann AR, Niimi A, Ogi T, Brown S, Sabbioneda S, et al (2007) Translesion synthesis: Y-family polymerases and the polymerase switch. DNA Repair (Amst) 6: 891–899. 17363342 10.1016/j.dnarep.2007.02.003

[29] Despras E, Delrieu N, Garandeau C, Ahmed-Seghir S, Kannouche PL (2012) Regulation of the specialized DNA polymerase eta: Revisiting the biological relevance of its PCNA- and ubiquitin-binding motifs. Environ Mol Mutagen 53: 752–765. 23076824 10.1002/em.21741

[30] Schopf B, Bregenhorn S, Quivy JP, Kadyrov FA, Almouzni G, et al (2012) Interplay between mismatch repair and chromatin assembly. Proc Natl Acad Sci U S A 109: 1895–1900. 22232658 10.1073/pnas.1106696109 PMC3277549

[31] Uwada J, Tanaka N, Yamaguchi Y, Uchimura Y, Shibahara K, et al (2010) The p150 subunit of CAF-1 causes association of SUMO2/3 with the DNA replication foci. Biochem Biophys Res Commun 391: 407–413. 19919826 10.1016/j.bbrc.2009.11.071

[32] Punchihewa C, Inoue A, Hishiki A, Fujikawa Y, Connelly M, et al (2012) Identification of Small Molecule Proliferating Cell Nuclear Antigen (PCNA) Inhibitor That Disrupts Interactions with PIP-box Proteins and Inhibits DNA Replication. J Biol Chem 287: 14289–14300. 22383522 10.1074/jbc.M112.353201 PMC3340206

[33] Tan ZQ, Wortman M, Dillehay KL, Seibel WL, Evelyn CR, et al (2012) Small-Molecule Targeting of Proliferating Cell Nuclear Antigen Chromatin Association Inhibits Tumor Cell Growth. Mol Pharmacol 81: 811–819. 22399488 10.1124/mol.112.077735 PMC3362894

[34] Chan KS, Koh CG, Li HY (2012) Mitosis-targeted anti-cancer therapies: where they stand. Cell Death Dis 3: e411. 23076219 10.1038/cddis.2012.148 PMC3481136

